# *Caloparyphus palaearcticus* sp. n. (Diptera, Stratiomyidae), the first record for the soldier fly genus in the Palaearctic

**DOI:** 10.3897/zookeys.594.7750

**Published:** 2016-05-30

**Authors:** Rudolf Rozkošný, Martin Hauser, Jon K. Gelhaus

**Affiliations:** 1 Department of Botany and Zoology, Faculty of Science, Masaryk University, Kotlářská 2, 61137 Brno, Czech Republic; 2 California Department of Food and Agriculture, Plant Pest Diagnostics Branch, 3294 Meadowview Road, Sacramento, California, USA 95832-1448; 3 Department of Entomology, The Academy of Natural Sciences of Drexel University, 1900 Ben Franklin Parkway, Philadelphia, USA 19103-1195

**Keywords:** Palaearctic Region, Caloparyphus, Oxycerini, taxonomy, new species, Russia, Mongolia, soldier fly

## Abstract

*Caloparyphus palaearcticus* **sp. n.** is described from Russia and two localities in Mongolia and is the first representative of this genus in the Palaearctic and the only species found outside the New World. The morphological characters of the species are described and illustrated, and relationships to related species of *Caloparyphus* are discussed.

## Introduction

The genus *Caloparyphus* belongs to the subfamily Stratiomyinae and the tribe Oxycerini. James (1939) described it originally as a subgenus of *Euparyphus* Gerstaecker and later it was treated as a distinct genus by Quist and James (1973). Sinclair (1989) described madicolous larvae of *Caloparyphus greylockensis* (Johnson, 1912) and *Caloparyphus tetraspilus* (Loew, 1866), and added adult characters of some further species. Some species of *Caloparyphus* are still insufficiently known or based on the female holotypes with the relevant males described only superficially or not at all. Moreover, a precise identification is complicated by the known sexual dimorphism especially in the shape and the colour pattern of the head. According to the World Catalogue of the Stratiomyidae (Woodley 2001, 2011) twelve almost exclusively Nearctic species of *Caloparyphus* are known, with only *Caloparyphus decemmaculatus* (Osten Sacken, 1886) penetrating into Mexico.

Recently we obtained males and females from Palaearctic Asia (Russian Kamchatka and Mongolia) that undoubtedly belong to *Caloparyphus*. They display all the main diagnostic characters of the genus (apical flagellomere style-like, antennal pedicel not elongated, scutellar spines separated by a distance as great as the length of spines, abdomen black with yellow oblique lateral spots on tergites 3 and 4, aedeagus tripartite distally). We can thus prove the occurrence of *Caloparyphus* in the Palaearctic Asia and describe a new species.

## Methods

Part of the material was collected during the PIRE Mongolia project (http://mongolia.bio.upenn.edu), University of Pennsylvania (http://mongolia.bio.upenn.edu), and deposited in the collections of the Mongolian Aquatic Insect Survey (http://clade.acnatsci.org/mongolia) (Principal Investigator and Director of MAIS: Jon K. Gelhaus), the latter a project to document the Mongolian aquatic invertebrate diversity with respect to evolution, ecology and water quality (http://clade.ansp.org/entomology/mongolia/mais_home.html). More than 600 specimens of Mongolian Stratiomyidae have been examined and identified through the MAIS project and results are being prepared for publishing.

The notation in brackets for Arkhangai Mongolia paratypes refers to their map coordinates on the Mongolian national government topographic map 47T

The examined specimens were studied with Olympus and Nikon SMZ 1500 Stereomicroscopes. Photographs were taken through a Canon 450D and a Nikon DS-5M camera and were edited by CombineZ, Helicon Focus and Adobe Photoshop CS 4 software. The terminalia of the examined specimens were macerated in 10% KOH, rinsed with water and then preserved in glycerin and placed in a microvial on the specimen pin.

Morphological terminology follows that of McAlpine (1981) as modified by Cumming and Wood (2009). Body lengths are given exclusive of antennae.

### Collection acronyms

ANSP
 The Academy of Natural Sciences of Drexel University, Philadelphia, PA, USA 


CSCA
California State Collection of Arthropods, Department of Food and Agriculture, Sacramento, CA, USA 


USNM
Department of Entomology, Smithsonian Institution, Washington DC, USA 


## Taxonomy

### Caloparyphus palaearcticus

Taxon classificationAnimaliaDipteraStratiomyidae

Rozkošný, Hauser & Gelhaus sp. n.

http://zoobank.org/92DFC733-E84E-44E6-BEE7-18323D9F8247

Figs 1 , 2 , 3–6 , 7 , 8 , 10 , 11

#### Type material.

**Figure 1.**
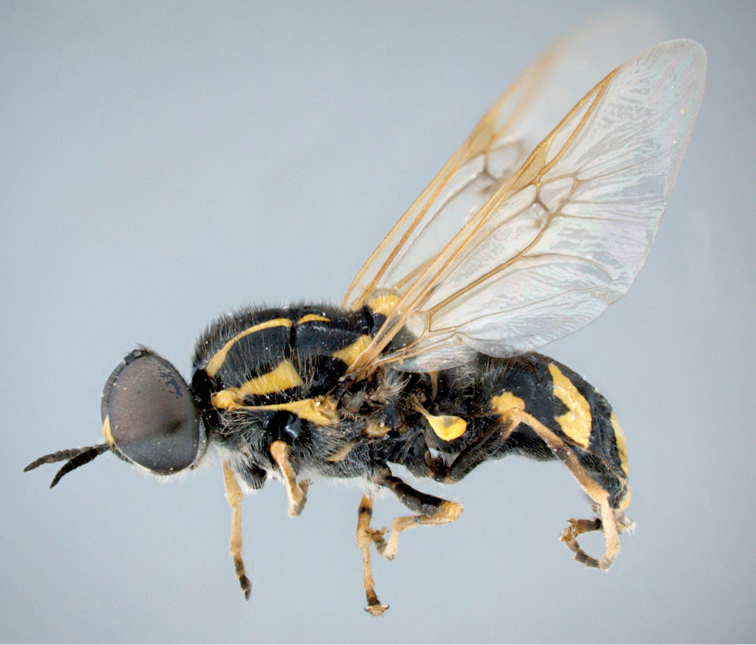
*Caloparyphus palaearcticus* sp. n. holotype, male, habitus lateral view.

**Figure 2.**
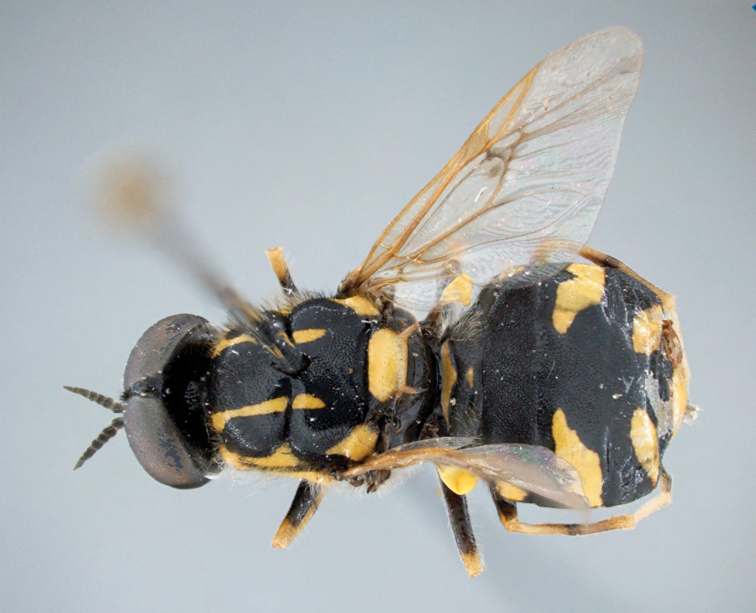
*Caloparyphus palaearcticus* sp. n. holotype, male, habitus dorsal view.

**Figures 3–6.**
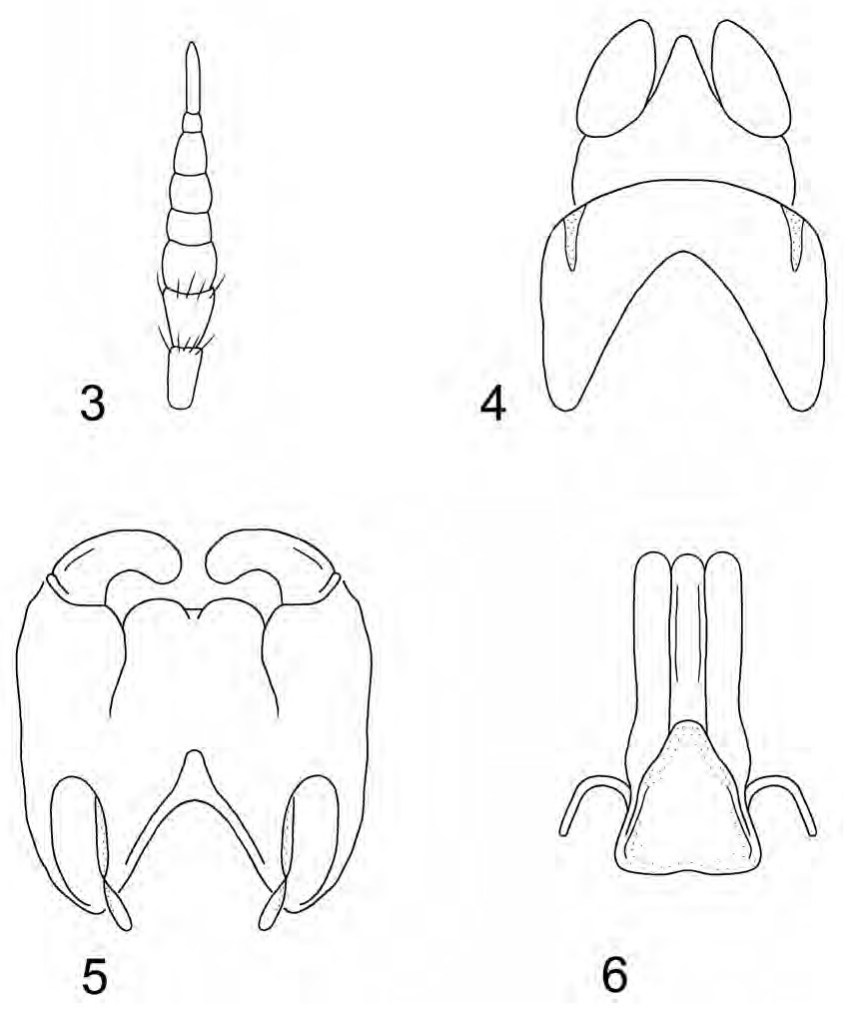
*Caloparyphus palaearcticus* sp. n. **3** Antenna **4** Epandrium **5** Genital capsule **6** Aedeagal complex.

Holotype male (Figs 1–6), **Russia**: Kamchatka, okrestnosti [=environs] ESSO, lesnaja doroga [=forest road], 4.vii.2008, V. Mutin (label in Cyrillic) (deposited in CSCA).

Paratypes: 1 male, **Mongolia**: Hövsgöl Aimag, Hövsgöl Nuur (lake), east shore area, Dalbay Gol (river) valley, 51°01'40.5"N, 100°45'60.0"E, 1670 m, 22.vii.2007, D. Song (ID 263) (deposited in ANSP). 3 females, **Mongolia**: 3 females, Mongolia: Arkhangai Aimag, 17 km SW Tsenher, 1820m, swamp along stream, ca. 47°21'N, 101°33'E [47T 698.051 5243.562], 21.vii.2014 A.v.Eck (deposited in CSCA and USNM).

#### Diagnosis.

**Figure 7.**
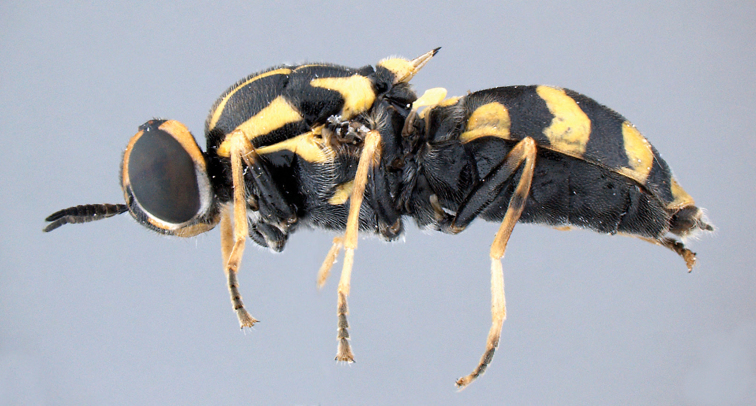
*Caloparyphus palaearcticus* sp. n. paratype, female, habitus lateral view.

*Caloparyphus palaearcticus* is the only species in the genus found in the Palaearctic Region. The males can be easily distinguished from all the other species of this genus by the two distinct brownish spots of denser microtrichia on the wing membrane (Fig. 1) in the lower distal corner of the basal medial cell and in the middle at the lower margin of the discal cell. In both sexes the antennae are as long as the head (Figs 2, 7), and in the wing, vein R4 is absent and the discal cell is completely covered with microtrichia and has denser microtrichia patches in the males (Fig. 10).

**Figure 8.**
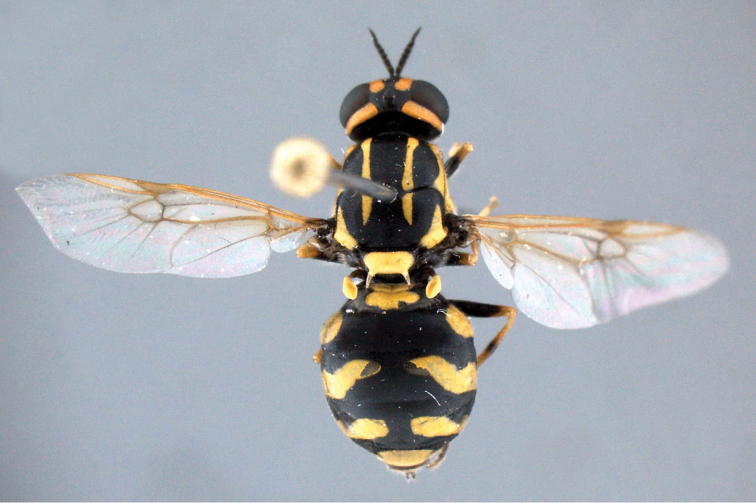
*Caloparyphus palaearcticus* sp. n. paratype, female, habitus dorsal view.

**Figures 9–10.**
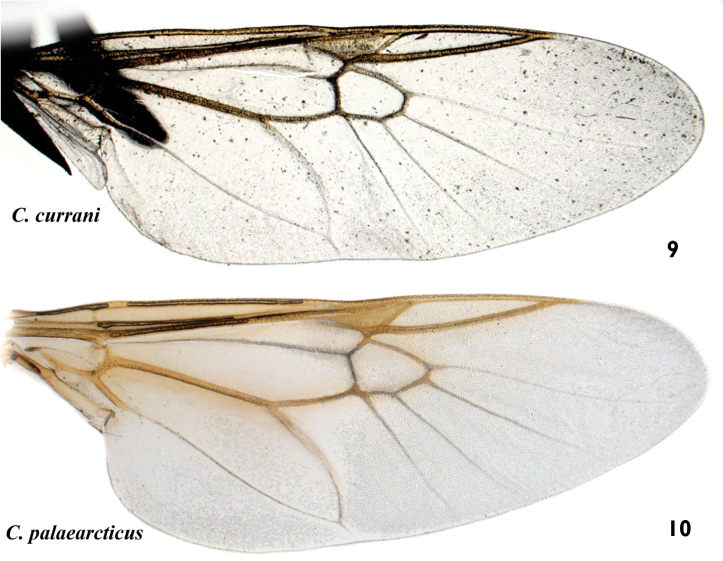
*Caloparyphus* spp. wing. **9** *Caloparyphus currani*, holotype **10** *Caloparyphus palaearcticus* sp. n., paratype, female.

#### Description.

**Male.** (Figs 1–6) *Head*: rounded, 1.3 times higher than long in profile and 1.6 times broader than high in frontal view. Compound eyes touching in middle third of frons and consisting of larger facets on greater than lower half of its surface. Distinct border between differentially large facets and smaller facets at level of antennal insertion. Ocellar triangle slightly longer than wide, prominent in lateral view. Upper frons barely twice as wide as anterior ocellus. Lower frons somewhat broader than long, predominantly shining yellow but small upper corner black. Antenna (Fig. 3) about as long as head in profile, relative length of antennal segments scape: pedicel: flagellomere 1: flagellomere 2: flagellomere 3: flagellomere 4: flagellomere 5: flagellomere 6 as 2.7: 2.5: 2.4: 1.8: 1.8: 2.4: 1:0: 2.6. Face shining black with narrow whitish lateral stripes along inner eye margin reaching yellow frontal spots and dilated in lower half of face. Proboscis yellow with shining black theca, black palpus very short. Ventral part of head and postgenal area black but latter grayish dusted. Head pile mostly inconspicuous, pale yellowish to white, hairs on occipital margin black.

*Thorax*: Shining black with two pairs of bright yellow scutal vittae. Dorsal vittae dilated in anterior third and reaching beyond transverse suture. Each lateral vitta touching yellow postpronotal callus anteriorly and transverse suture posteriorly. Also postalar callus intensively yellow, with a pointed anterior projection. Scutellum yellow but its narrow base and lateral parts black, scutellar spines yellow but blackish distally. Pleural part of thorax predominantly shining black, yellow line along upper margin of anepisternum abruptly dilated in front of wing base. Katepimeron and upper posterior part of katepisternum contrastingly yellow. Thoracic pile moderately long, mainly whitish but black and upright on scutum though similar whitish hairs on anterior part and along notopleura also visible. Wing membrane hyaline, veins brownish to pale yellow, stigma yellowish. No vein arising from discal cell reaching wing margin. Wing microtrichia considerably reduced in basal half of wing membrane, limited to small distal areas in basal radial and basal medial cells, sparse microtrichia in central area of posterior cubital cell and distal half of anal cell. Apical half of wing membrane almost completely covered with dense microtrichia but anterior cubital cell bare along upper and inner margins. Especially dense microtrichia visible at distal part of basal medial cell and along anterior lower corner of discal cell as two distinct darkened microtrichial patches (cf. Figs 1 and 2). Calypters inconspicuous, dark, with long and upright whitish hairs. Halteres yellow, only the stem darkened basally. Legs black and yellow: coxae black, femora black with yellow tips, tibiae yellow with darkened ring on mid and hind pairs, tarsi predominantly darkened, only all basitarsi yellow. Pile on legs mainly short, whitish to yellow, often predominantly black on darkened parts, pile on basal halves of femora longer.

*Abdomen*: About as long as broad, sub-circular, black with yellow pattern (Fig. 1), venter black. Abdominal pile short, black and mostly semi-appressed, longer hairs visible in anterior corners, yellow spots bare. Ventral hairs whitish and mostly appressed.

*Terminalia*: Simple, without distinct modifications. Epandrium with a membranous incision before posterior corner on each side distally, proctiger subtriangular and cerci relatively short, oval (Fig. 4). Genital capsule (Fig. 5) with two rounded medial lobes distally and aedeagal complex (Fig. 6) simple, tripartite.

*Length*: body 6.5–6.7 mm, wing 5.7–5.8 mm.

**Figures 11–12.**
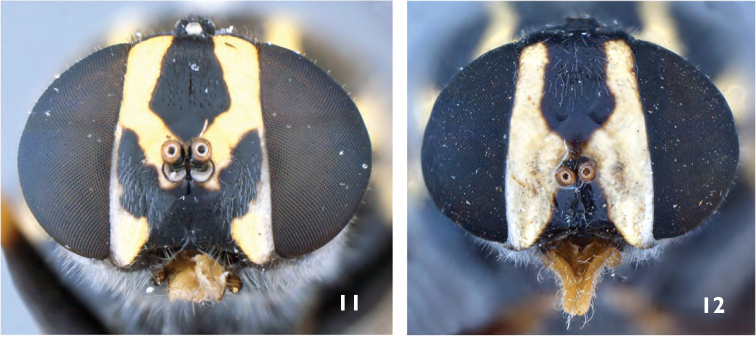
*Caloparyphus* spp. female head. **11** *Caloparyphus palaearcticus* sp. n., paratype, female **12** *Caloparyphus currani*, holotype.

**Female.** Similar to male, except for typical sexual dimorphism. Face yellow (Fig. 11) with a dark pentagonal mark on the frons widening from the ocellar triangular towards the antennal base, and narrowing on the lower frons before reaching the antennae. Area around the mouth black, and extending laterally upwards to the eye margin beyond antennae. Lower half of eye margin with a narrow vitta of dense silver pubescence. Hairs on lower face white, and black on frons. Gena and occiput mainly yellow, black around ocellar triangle, this black area extends to the upper eye margin and is also connected to the black pentagonal mark on the frons. The yellow spot on tergite one (Fig. 8) is more extant than in the male, also the lateral spots on tergite two are larger and more rounded (Fig. 7).

*Length*: body 7.9–8.0 mm, wing 6.9–7.0 mm.

#### Variability.

There are no doubts that the male holotype and paratypes are conspecific but some small differences in color pattern were found in the male paratype from Mongolia (e.g. dorsolateral vittae are separated from the yellow postpronotal calli and a spot at the katepisternum and a small yellow basal spot on the abdomen are missing). Similar variability is commonly known in many other Oxycerini. Differences in color pattern between the female and male adults are noted in the description.

#### Etymology.

The species epithet indicates the distribution of this species in the Palaearctic Region, i.e. in a different biogeographic realm in comparison with all other known species of this genus.

#### Distribution.

Eastern part of the Palaearctic Region from Mongolia to Russian Kamchatka (Fig. 14).

#### Ecology.

**Figure 13.**
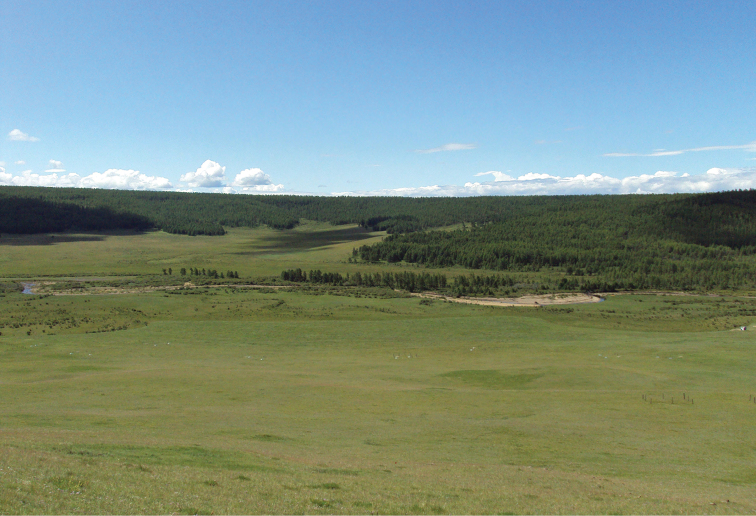
Habitat of *Caloparyphus palaearcticus* sp. n. paratype, Mongolia, Hövsgöl Aimag, Hövsgöl Nuur (lake), Dalbay Gol (river) valley.

The male Mongolian specimen was collected in an area of mixed steppe grassland, riparian shrubs and *Larix siberica* forest (Fig. 13). Insects were collected visiting flowers on the south facing slope of the steppe dominated by a mixture of sedges (e.g. *Carex* sp.), grasses (e.g. *Festuca lenensis*, *Poa attenuata*) and forbs (e.g. *Aster alpinus*, *Potentilla* spp., *Artemisia commutata*, *Thymus gobicus*). Further information about the study site can be found in Song (2015).

**Figure 14.**
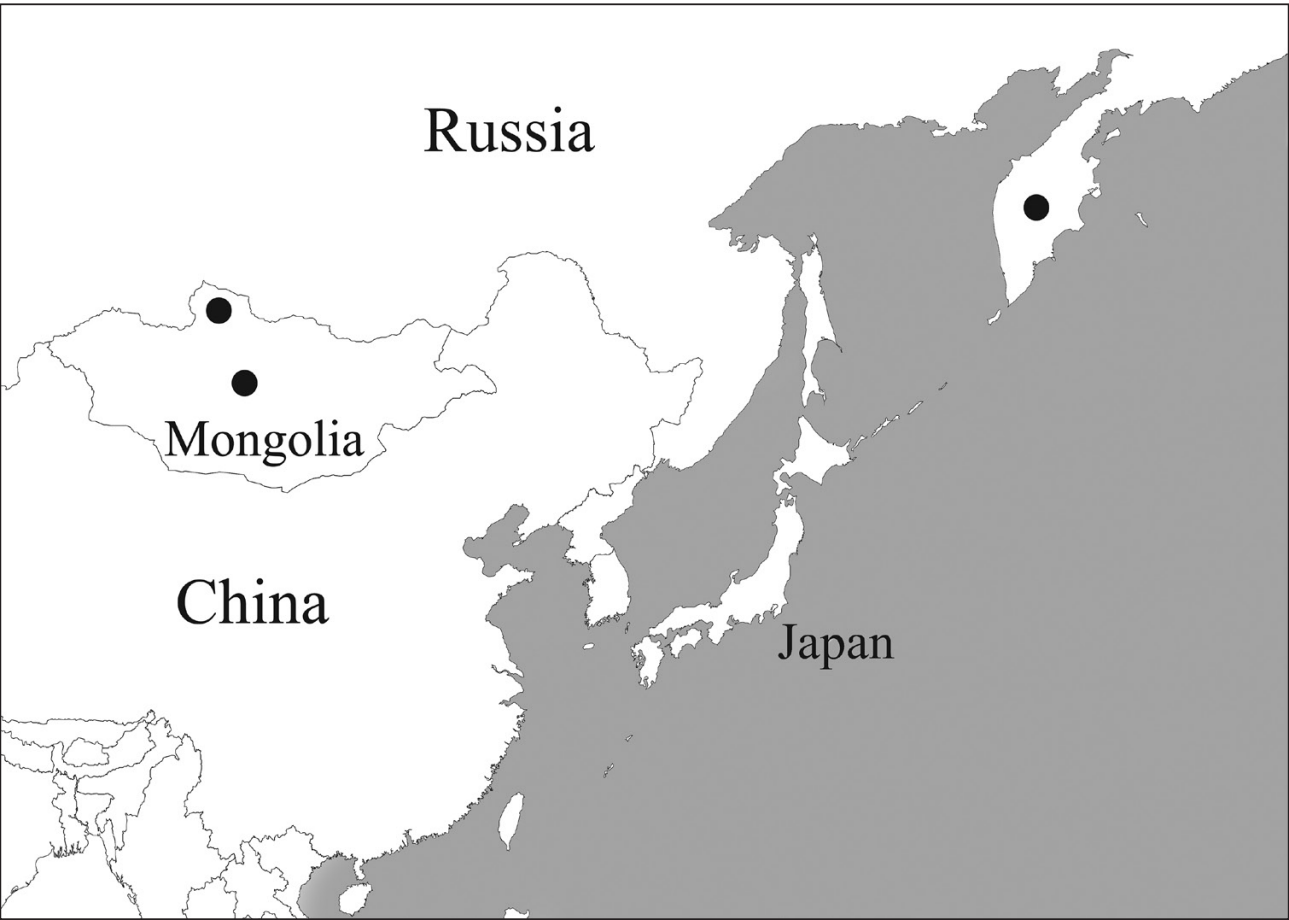
Distribution map of *Caloparyphus palaearcticus* sp. n.

## Discussion

The species of the genus *Caloparyphus* were only revised by James (1939), when he erected “the species of *Euparyphus* related to *crotchii*” as a subgenus of *Euparyphus*. In that paper three species considered today part of the genus were not included: *Caloparyphus tetraspilus*, *Caloparyphus atriventris* (Coquillett, 1902), and *Caloparyphus greylockensis*.

Of the twelve valid species of *Caloparyphus* (Woodley 2001) the following types have been examined by M. Hauser: *Caloparyphus currani* (James, 1939), *Caloparyphus flaviventris* (James, 1932), *Caloparyphus mariposa* (James, 1939), and Norm Woodley: *Caloparyphus amplus* (Coquillett, 1902), *Caloparyphus atriventris*, *Caloparyphus crucigerus* (Coquillett, 1902), and *Caloparyphus tahoensis* Coquillett, 1902 (synonym to *Caloparyphus crucigerus*). Most other species have been studied using specimens from several collections and comparing with the type series of the new species. The following differences might help in distinguishing this new Palaearctic species from the Nearctic species:

*Caloparyphus decemmaculatus*: The scape and pedicel distinctly elongated and tergites 2–4 have central spots (this species might not belong into this genus);

*Caloparyphus tetraspilus* has a black scutellum as well as four, two or no central spots on tergites 3-4 and no extended lateral markings on the tergites;

*Caloparyphus crotchi* (Osten Sacken, 1877), *Caloparyphus flaviventris*, *Caloparyphus major* (Hine, 1901), *Caloparyphus mariposa* and *Caloparyphus pretiosus* (Banks, 1920) have the antennae distinctly longer than the head and vein R_4_ present;

*Caloparyphus greylockensis* has no or very short vittae on the mesonotum;

*Caloparyphus crotchi* has the scape twice as long as the pedicel, the mesonotal vittae ending at the suture and the male has the hind metatarsus at the apex enlarged;

*Caloparyphus amplus* has the wing mainly bare (especially cell d) and vein R_4_ present;

*Caloparyphus atriventris*, *Caloparyphus currani* and *Caloparyphus crucigerus* have the wing mainly bare, especially the discal cell which is devoid of microtrichia.

The species which seem to be most similar to *Caloparyphus palaearcticus* sp. n. are the members of the *crucigerus*-group (*Caloparyphus crucigerus*, *Caloparyphus atriventris*, *Caloparyphus currani*) and within it especially *Caloparyphus currani*. But the yellow coloration of the face of these two species (Figs 11–12) is distinctly different. Although the extent of the yellow coloration shows variation in other species of this genus, it is constant in all the female paratypes of *Caloparyphus palaearcticus* sp. n. and there is no other specimen known besides the female holotype for *Caloparyphus currani*. The major differences next to the coloration of the head is that the apical half and most of the posterior portion of the wing in *Caloparyphus palaearcticus* sp. n. (Fig. 10) is covered with microtrichia, especially the discal cell and cell r_4+5_, the apical part of cell br and the apical half of cell cup, with most of these areas bare in *Caloparyphus currani* (Fig. 9). The dark coloration on the femora and the apical segments of the tarsi are much darker in *Caloparyphus palaearcticus* sp. n. (Figs 1, 7) than in *Caloparyphus currani*.

It is remarkable that there is no other specimen of *Caloparyphus currani* found so far except the holotype. The holotype might be just a large specimen of another described species in the *crucigerus*-group. The Nearctic species of the *crucigerus*-group need to be revised, as there are several potential new species, one in southern California, and one in Canada, and the status of *C tahoensis*, which is currently a synonym of *Caloparyphus crucigerus*, should be reexamined. But this is beyond the scope of this publication, in which we wanted show that the only Palaearctic species is distinct from all described Nearctic taxa.

This disjunct distribution of *Caloparyphus palaearcticus* sp. n. is similar to other insects found in northern Mongolia and the Russian Far East. For example, Gelhaus and Podenas (2006) in a study of crane flies (Tipuloidea) from the same Lake Hövsgöl watershed noted that those species with a disjunct distribution in northern Mongolia and the Russian Far East comprised 9.4% of the total crane fly fauna, or 8 out of 85 species found. A similar disjunction was again recently noted where a new species was found in the crane fly genus *Heterangeus* in northern Mongolia (Hentiy mountains); the genus was known previously only from the Russian Far East, Korean peninsula and Japan (Podenas, Podeniene and Gelhaus, 2014).

## Supplementary Material

XML Treatment for Caloparyphus palaearcticus
